# The Length of Leukocyte and Femoral Artery Telomeres in Patients with Peripheral Atherosclerosis

**DOI:** 10.3390/genes13040704

**Published:** 2022-04-15

**Authors:** Ewa Boniewska-Bernacka, Anna Pańczyszyn, Jacek Hobot, Piotr Donizy, Zbigniew Ziembik, Anna Goc, Marian Klinger

**Affiliations:** 1 Medical Department, Institute of Medical Sciences, University of Opole, ul. Oleska 48, 45-052 Opole, Poland; apanczyszyn@uni.opole.pl (A.P.); j.hobot@uni.opole.pl (J.H.); anna.goc@uni.opole.pl (A.G.); marian.klinger@uni.opole.pl (M.K.); 2 Department of Clinical and Experimental Pathology, Wroclaw Medical University, 50-556 Wroclaw, Poland; piotrdonizy@wp.pl; 3 Institute of Environmental Engineering and Biotechnology, University of Opole, 45-032 Opole, Poland; ziembik@uni.opole.pl

**Keywords:** telomere length, atherosclerosis, cholesterol, inflammation markers

## Abstract

The length of telomeres (TLs) that protect chromosome ends may reflect the age of cells as well as the degree of genetic material damage caused by external factors. Since leukocyte telomere length is associated with cardiovascular diseases, the aim of this study was to evaluate whether leukocyte TL reflects femoral artery wall telomeres of patients with atherosclerosis and lower limb ischemia. Samples of femoral artery wall and blood were collected from 32 patients qualified to surgical revascularization. The analysis included blood and artery wall telomere length measurement and biochemical parameters. The study indicated that there was a moderate correlation between artery wall TL and leukocyte TL. Leukocyte TL was, on average, two times shorter than artery wall TL and correlated with the number of white blood cells. In turn, artery TL was impacted by total cholesterol level. The results suggest that the length of leukocyte telomeres may reflect artery wall TL and indirectly reflect the processes taking place in the artery wall in patients with atherosclerosis.

## 1. Introduction

At the end of each chromosome, there are telomeres that are composed of a six-nucleotide sequence and proteins. The main role of telomeres is to protect chromosomes and provide genetic stability [1]. The progressive shortening of telomeres over time is a result of DNA replication during cell division [2]. Changes in their length have been used as a common indicator for cellular senescence and aging [3]. Interestingly, mounting evidence in the literature suggests that telomere length (TL) may be associated with cardiovascular diseases (CVDs) and their risk factors [4]. However, it is not clear whether the link between shorter telomeres and CVD implies a causal relationship [5].

A high cholesterol level is one of the risk factors for cardiovascular diseases. It causes oxidative stress, which leads to telomere shortening and cellular senescence as well as the development of atherosclerotic plaque. The plaque deposition causes damage to the vascular endothelium and leads to proliferation of hematopoietic progenitor cells (HSCs) that induce an increase in leukocytes. Leukocytes during inflammatory response release more reactive oxygen species (ROS), aggravating the pathological condition. Thus, the length of telomeres may reflect the effect of oxidative stress, inflammation and mechanical stress in vascular cells [4,6,7].

Leukocyte TL from peripheral blood was studied as a potential indicator of diseases related to age, such as CVD [8], atherosclerosis [9] and hypertension [10]. Population prospective studies demonstrated that individuals with short leukocyte TLs were more vulnerable to being subject to cardiovascular events, stroke and mortality [11,12,13]. In turn, the works of other authors did not prove those relationships. For example, De Mayer et al. [14] and Rietzschel et al. [15] did not find any association between leukocyte TL and atherosclerosis. Other studies have shown that individuals, depending on the degree of atherosclerosis advancement, have significantly variable telomere lengths in their leukocytes [16]. Therefore, the literature data seem to be divergent. Although it is widely believed that leukocyte TL corresponds with other tissue TL [17], it may not directly reflect the local pathological process. The present study was conducted with the aim of determining if there is any relationship between artery wall TL and leukocyte TL in patients with peripheral arterial disease. An important aspect of this study was to examine whether leukocyte TL may reflect local pathological lesions of the artery. Another goal of this work was to analyze possible factors, including inflammation markers, that may have an impact on artery and leukocyte TL.

## 2. Materials and Methods

### 2.1. Patients Samples

The study involved 32 patients (30 men and 2 women) treated at the University Clinical Hospital in Opole for lower limb ischemia in the course of critical stenosis/occlusion of the femoral artery division. In all patients, femoral artery endarterectomy and profundoplasty were performed. The femoral artery was reconstructed with a saphenous vein patch. They also underwent a medical examination and provided venous blood and tissue samples.

This research project was approved by the local ethics committee (KB/52/No2/2019). All patients were informed about the aim of the study, and each participant gave their written informed consent and completed the information survey.

### 2.2. DNA Preparation and Quantitative Polymerase Chain Reaction (qPCR)

DNA from venous blood and from the femoral artery was isolated using the GeneMATRIX Quick Blood DNA Purification Kit and the GeneMATRIX Tissue DNA Purification Kit (Eurx, Poland), respectively, according to the manufacturer’s instructions. Isolated DNA was then quantified for further analysis using a BioSpectrometer (Eppendorf, Germany). 

A quantitative PCR reaction was applied to determine telomere length according to O’Callaghan and Fenech [18] with minor modifications. The synthetic oligomers for albumin and telomeres were diluted (10 to 0.001 pg for telomere oligomers and 1 to 0.0001 pg for albumin oligomers) (Table 1) and used as a reference sample to prepare the standard curve. Each plate included a standard curve. Plates for telomeres and the reference gene (albumin) were run separately. The results were calculated only when the efficiency of both reactions was equal [19]. All experimental and standard samples were run in triplicate. Each reaction well contained 2 µL of DNA (10 ng), 2× SsoAdvanced Univ SYBR Grn Supr (Bio-Rad), telomere primers (i.e., TeloF and TeloR primers—500 nM) or primers for albumin (Albu and Albd primers—500 nM) and water to a final volume of 10 µL. The thermal cycling profile for telomeres was as follows: 10 min at 95 °C, 30 cycles for 15 s at 95 °C and 1 min at 60 °C with a signal acquisition; for albumin: 15 min at 95 °C, 2 cycles for 15 s at 94 °C and 15 s at 49 °C and 35 cycles for 15 s at 94 °C, 30 s at 84 °C and 15 s at 85 °C with a signal acquisition. The standard curve for each plate was generated after thermal cycling and raw data collection by CFX Manager Software (Bio-Rad). The efficiency of the reaction was equal for telomeres and albumin, and it was not lower than 90%. The variation of Ct values in the sample was <0.5 Ct (SD < 0.25) in both the telomere and albumin runs. Mean Ct values were used to calculate the absolute telomere length in bp, as described in [18].

### 2.3. Statistical Analysis

Data were analyzed using R software, version 3.5.1 Vienna, Austria, (http://cran.r-project.org accessed on 1 January 2020). Nominal variables are presented as *n* (%) while continuous variables as the mean ± SD or median (Q1; Q3), depending on the distribution. The data distribution normality was assessed using the Shapiro–Wilk test and based on a visual assessment of histograms. Group comparison was carried out with ANOVA analysis. Additionally, univariate regression was used to model the level of leukocyte telomere length and artery wall telomere length. Separate models were prepared for leukocyte telomere length and artery wall telomere length. Models assessment included *R*^2^ and adjusted *R*^2^ levels. Additionally, the correlation between leukocyte TL and artery wall TL was assessed using Pearson’s correlation and Pearson’s partial correlation controlling for the effect of white blood cells (WBCs). All tests were based on α = 0.05.

## 3. Results

The study included 32 patients (30 men and 2 women) of the University Clinical Hospital of Opole. The average age was 67.6 ± 8.36 years. Detailed results of the biochemical test assessments are included in Table 2.

**Figure 1:**
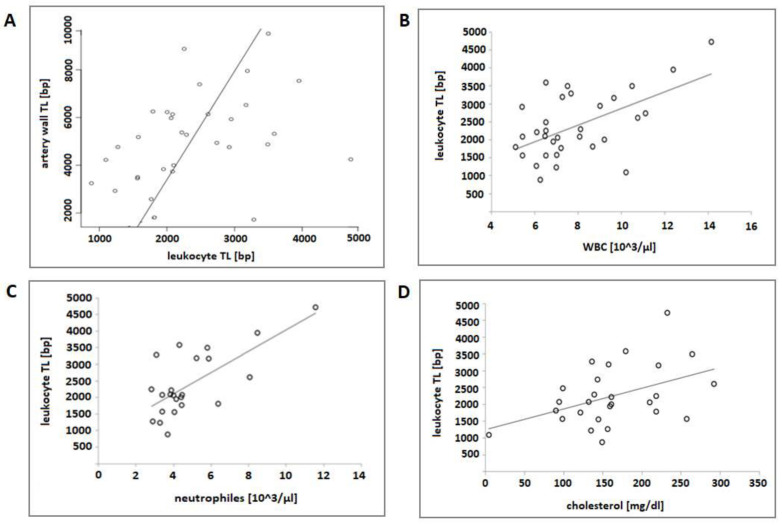
Correlation between leukocyte TL and (**A**) artery wall TL (Pearson’s correlation, *R* = 0.3751; *p* = 0.034); (**B**) WBCs (Pearson’s correlation, *R* = 0.5608; *p* = 0.0008); (**C**) neutrophils (Pearson’s correlation, *R* = 0.6985; *p* = 0.0002); (**D**) total cholesterol (Pearson’s correlation, *R* = 0.5093; *p* = 0.049).

There was a significant positive correlation between artery wall TL and leukocyte TL (*R* = 0.38, *p* = 0.034) (Figure 1A). The analysis indicated that artery wall telomeres were, on average, 2.34 ± 0.88 times longer than leukocyte telomeres (Table 2). There was a tendency towards leukocyte telomere shortening according to age. A weak negative correlation between age and leukocyte telomeres and artery wall TL was present but without statistical significance (*p* = 0.056 for leukocyte TL and *p* = 0.11 for artery TL). A trend towards a higher count of WBCs and neutrophils with increasing telomere length was observed (Figure 1B,C) as well as a positive correlation between leukocyte TL and total cholesterol (Figure 1D).

In univariate models, the length of leukocyte telomeres was significantly positively impacted by cholesterol level, β = 3.62 (95% CI: 0.82 to 6.42, *p* = 0.013); WBC count, β = 232.03 (95% CI: 4.30 to 359.76, *p* = 0.001); neutrophils, β = 320.42 (95% CI: 171.45 to 469.39, *p* < 0.001) (Table 3).

**Figure 2:**
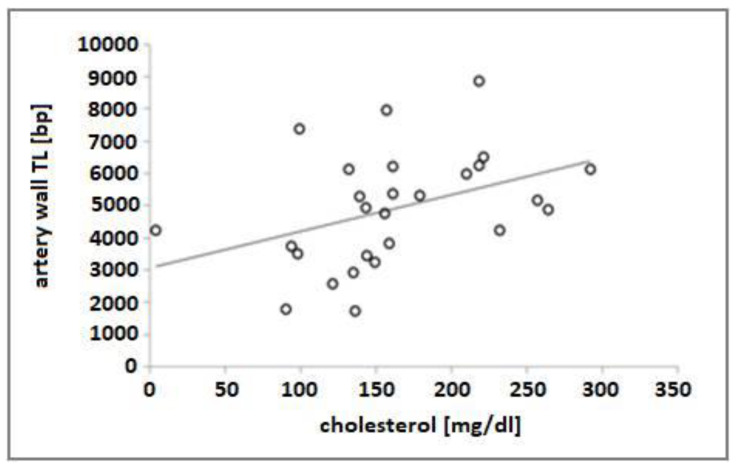
Correlation between artery wall TL and total cholesterol (*Pearson’s correlation, R = 0.6985; p = 0.0002*).

The length of artery wall telomeres in univariate models was significantly positively impacted only by cholesterol level, β = 6.68 (95% CI: 0.87 to 12.48, *p* = 0.026) (Table 4, Figure 2).

## 4. Discussion

Telomeres play an important protective role against premature cell aging. Their progressive shortening and dysfunction lead to cell senescence.

According to global epidemiological statistics, the main population causes of mortality are CVDs [21,22]. In the case of CVDs, including atherosclerosis, studies indicated that permanent DNA damage occurs in the telomeric regions as a result of mitochondrial dysfunction. This phenomenon is independent of cell proliferation and telomere length [23]. Hence, telomeres have been extensively studied in order to figure out whether their length may be useful as cardiovascular diseases prognostic factor. Several studies demonstrated that shorter telomeres of leukocytes positively correlate with a higher risk of CVD [24].

The aim of the current study was to examine whether there is any relation between leukocyte and artery wall. To our knowledge, this is the first study in which artery wall telomeres of patients with lower limb ischemia were analyzed together with leukocyte telomeres. In previous studies [2,17,25], high TL variability was observed among different tissues as well as between individuals. A study of twelve different cadavers’ tissues showed the highest TL in peripheral leukocytes and a significant correlation only between leukocyte, muscle and liver telomere length [2]. However, in comparison to TL from living donors, both Daniali et al. [17] and Demanelis et al. [25] reported that leukocytes have the shortest telomeres. According to the authors, telomere length was positively correlated across human tissue types, and leukocyte TL can serve as a proxy for tissue-specific telomere length. Since previous studies included healthy tissues, it is uncertain whether leukocyte TL correlated with the TL of tissues affected by the pathological process.

In our study, leukocyte TL was shorter (on average 2.34 ± 0.88 times) than for artery wall. Similarly, Nzietchueng et al. [26] also indicated that arterial segments, both with and without atherosclerosis lesions, had longer telomeres in comparison with leukocyte telomeres. We also observed variances between individuals. Nevertheless, there was a moderate positive correlation between leukocyte and artery wall TLs, which is consistent with previous findings. On account of the large difference between leukocyte and artery TLs, we used a univariate regression model to evaluate parameters that may affect leukocyte and artery telomere length.

We observed a positive correlation between leukocyte TL and WBC numbers, especially including neutrophils, that complies with previously published results [27,28]. The association between telomere length and complete blood count has been reported in the literature. A significant positive relationship was found with the number of white blood cells and red blood cells, hemoglobin and hematocrit [29]. In addition, longer telomeres in blood cells were correlated with an increased number of RBC and WBC [30]. In contrast, De Meyer et al. [14] showed such an association for RBC, but not for WBC, both in men and women of middle age. Interestingly, some works did not prove any significant association between telomere length and complete blood count in elderly individuals [31,32,33]. The lack of association between telomere length and hematological parameters in elderly people may be explained by the fact that in hematopoietic stem cells of adults, a severe loss of telomere DNA was observed [34].

Our results support the concept that telomere attrition may be a biomarker for reduced proliferation reserve in hematopoietic progenitor cells, especially since our donors were over 60 years old [29,30].

In our univariate model, leukocyte TL was impacted by total cholesterol which is partially consistent with previous reports. Chen et al. [35,36] and Rehkopf et al. [37] demonstrated that there is a positive correlation between leukocyte TL and HDL-cholesterol. Because our data on LDL-cholesterol and HDL-cholesterol were limited to a few participants, we can only confirm the correlation between total cholesterol and leukocyte TL. Previous studies indicated that pathways of lipid synthesis and lipid uptake are activated in HSC. However, they become overactivated during inflammation including atherosclerosis [38]. Moreover, reduced lipoprotein uptake related to LDL shortage was associated with a lower level of hematopoietic precursors resident in the bone marrow together with cholesterol accumulation impacts on HSCs [39].

Our results showed that apart from total cholesterol, the other biochemical parameters did not affect artery telomere length. As mentioned, a positive correlation between leukocyte TL and HDL cholesterol was already observed [35,36], but there was no evidence that the same correlation exists in the case of artery wall telomeres and cholesterol. One of the possible explanations for this phenomenon may be the fact that all of the participants in the study were treated with statins. It is known that statins, which are traditionally taken in order to decrease cholesterol levels, display pleiotropic effects, and by modulating telomerase activity affect telomere erosion along with aging. Experiments have indicated that therapy with statins is associated with the activity of telomerase and lower telomere erosion, which seems to be a result of decreasing, either directly or indirectly, oxidative telomere DNA damage [40,41,42]. The limitation of our study was that it had a small number of patients, but the strength of this research was in the fact that the study group was quite homogenous. Furthermore, each patient was a kind of control for themselves, as we compared his/her length of artery wall telomeres with leukocyte TL.

## 5. Conclusions

In summary, our study showed, for the first time, that femoral artery wall TL is significantly longer than leukocyte TL in patients with peripheral atherosclerosis. The cholesterol level impacted both leukocyte and artery wall TL. A positive correlation between WBC and TL was observed only in the case of leukocytes, which suggests that different factors may have impacted leukocyte TL than for artery wall telomeres. The observed correlation between artery wall and leukocyte TL prompts the conclusion that leukocyte TL may reflect processes that take part in the artery wall of patients with atherosclerosis. The question of whether TL may be used as a prognostic marker of atherosclerosis severity remains open. Further studies with a large group of participants are needed to provide more detailed information.

## Figures and Tables

**Table 1:** Sequence of primers and oligomers used in the qPCR.

Primer/Oligomer	Sequence	Reference
Primer TeloF	CGGTTTGTTTGGGTTTGGGTTTGGGTTTGGG TTTGGGTT	[18]
Primer TeloR	GGCTTGCCTTACCCTTACCCTTACCC TTACCCTTACCCT	
Primer Albu	CGGCGGCGGGCGGCGCGGGCTGGGCGGaaatgctgcacagaatccttg	[20]
Primer Albd	GCCCGGCCCGCCGCGCCCGTCCCGCCGgaaaagcatggtcgcctgtt	
Oligomer tel	TTAGGGTTAGGGTTAGGGTTAGGGTTAGGGTTAGGGTTAGGGTTAGGGTTAGGGTTAGGGTTAGGGTTAGGGTTAGGG	[18]
Oligomer alb	CAGAGTCACCAAATGCTGCACAGAATCCTTGGTGAACAGGCGACCATGCTTTTCAGCTCTGGAA	[19]

**Table 2:** Characteristics of the study group.

Variable	*n* Available	Mean ± SD/Median (Q1; Q3)
Age, years	32	67.63 ± 8.36
Leukocyte telomeres	32	2381.63 ± 897.96
Artery wall telomeres	32	5131.72 ± 1 884.15
Ratio (artery wall TL/leukocyte TL)	32	2.34 ± 0.88
Glucose, mg/dL	28	108.50 (95.25; 133.75)
Cholesterol, mg/dL	27	157.00 (135.50; 218.00)
CRP, mg/L	31	3.05 (1.30; 8.27)
LDL, mg/dL	9	58.20 (51.70; 95.00)
WBCs, 10^3^/uL	32	7.86 ± 2.17
HGB, g/dL	32	13.50 (12.25; 14.70)
Neutrophils, 10^3^/µL	23	4.14 (3.55; 5.52)
Lymphocytes, 10^3^/µL	23	2.09 ± 0.72
Platelets, 10^3^/µL	32	239.22 ± 73.48

**Table 3:** Univariate linear regression for leukocyte telomere length.

	β	95% CI	Std. β	*p*
Age, years	−25.44	−64.36 to 13.49	−0.24	0.192
Glucose, mg/dL	4.52	−0.70 to 9.74	0.34	0.087
**Cholesterol, mg/dL**	**3.62**	**0.82 to 6.42**	**0.47**	**0.013**
CRP, mg/L	18.02	−15.98 to 52.03	0.20	0.287
LDL, mg/dL	−5.10	−18.81 to 8.60	−0.31	0.408
**WBCs, 10^3^/uL**	**232.03**	**104.30 to 359.76**	**0.56**	**0.001**
HGB, g/dL	−8.80	−23.49 to 5.89	−0.22	0.231
**Neutrophils, 10^3^/uL**	**320.42**	**171.45 to 469.39**	**0.75**	**<0.001**
Lymphocytes, 10^3^/uL	116.30	−486.46 to 719.12	0.09	0.692
Platelets, 10^3^/uL	2.68	−1.77 to 7.12	0.02	0.229

β—beta coefficient in the regression model; Std. β—standardized beta; CI—confidence interval.

**Table 4:** Univariate regression for artery telomeres length.

	β	95% CI	Std. β	*p*
Age, years	−75.22	−154.46 to 4.02	−0.33	0.062
Glucose, mg/dL	−5.20	−17.15 to 6.54	−0.18	0.379
**Cholesterol, mg/dL**	**6.68**	**0.87 to 12.48**	**0.42**	**0.026**
CRP, mg/L	−6.24	−79.38 to 66.89	−0.03	0.863
LDL, mg/dL	5.93	−30.48 to 42.34	0.17	0.712
WBCs, 10^3^/uL	87.35	−234.71 to 409.40	0.10	0.584
HGB, g/dL	−18.15	−48.99 to 12.69	−0.21	0.239
Neutrophils, 10^3^/uL	153.50	−254.65 to 561.75	0.17	0.443
Lymphocytes, 10^3^/uL	855.20	−284.19 to 1994.60	0.33	0.134
Platelets, 10^3^/uL	5.17	−4.20 to 14.53	0.20	0.269

β—beta coefficient in regression model; Std. β—standardized beta; CI—confidence interval.

## Data Availability

The data sets supporting the conclusions of this article are included within the article.

## Abbreviations

TL—telomere length; CVDs—*cardiovascular diseases*; HSC—hematopoietic progenitor cell; ROS—reactive oxygen species.

## References

[1] Blackburn E., Epel E., Lin J. (2015). Human telomere biology: A contributory and interactive factor in aging, disease risks, and protection. Science.

[2] Dlouha D., Maluskova J., Kralova Lesna I., Lanska V., Hubacek J.A. (2014). Comparison of the relative telomere length measured in leukocytes and eleven different human tissues. Physiol. Res..

[3] Pańczyszyn A., Boniewska-Bernacka E., Goc A. (2020). The role of telomeres and telomerase in the senescence of postmitotic cells. DNA Repair.

[4] Boniewska-Bernacka E., Pańczyszyn A., Klinger M. (2020). Telomeres and telomerase in risk assessment of cardiovascular diseases. Exp. Cell Res..

[5] Hoffmann J., Richardson G., Haendeler J., Altschmied J., Andrés V., Spyridopoulos I. (2021). Telomerase as a Therapeutic Target in Cardiovascular Disease. Arterioscler. Thromb. Vasc. Biol..

[6] Sack M.N., Fyhrquist F.Y., Saijonmaa O.J., Fuster V., Kovacic J.C. (2017). Basic Biology of Oxidative Stress and the Cardiovascular System: Part 1 of a 3-Part Series. J. Am. Coll. Cardiol..

[7] Xu X., Hu H., Lin Y., Huang F., Ji H., Li Y., Lin S., Chen X., Duan S. (2019). Differences in Leukocyte Telomere Length between Coronary Heart Disease and Normal Population: A Multipopulation Meta-Analysis. Biomed. Res. Int..

[8] Yeh J.K., Wang C.Y. (2016). Telomeres and Telomerase in Cardiovascular Diseases. Genes.

[9] O’Donnell C.J., Demissie S., Kimura M., Levy D., Gardner J.P., White C., D’Agostino R.B., Wolf P.A., Polak J., Cupples L.A. (2008). Leukocyte telomere length and carotid artery intimal medial thickness: The Framingham Heart Study. Arterioscler. Thromb. Vasc. Biol..

[10] Morgan R.G., Ives S.J., Walker A.E., Cawthon R.M., Andtbacka R.H., Noyes D., Lesniewski L.A., Richardson R.S., Donato A.J. (2014). Role of arterial telomere dysfunction in hypertension: Relative contributions of telomere shortening and telomere uncapping. J. Hypertens..

[11] Stefler D., Malyutina S., Maximov V., Orlov P., Ivanoschuk D., Nikitin Y., Gafarov V., Ryabikov A., Voevoda M., Bobak M. (2018). Leukocyte telomere length and risk of coronary heart disease and stroke mortality: Prospective evidence from a Russian cohort. Sci. Rep..

[12] Yin H., Akawi O., Fox S.A., Li F., O’Neil C., Balint B., Arpino J.M., Watson A., Wong J., Guo L. (2018). Cardiac-Referenced Leukocyte Telomere Length and Outcomes After Cardiovascular Surgery. JACC Basic Transl. Sci..

[13] Xu C., Wang Z., Su X., Da M., Yang Z., Duan W., Mo X. (2020). Association between leukocyte telomere length and cardiovascular disease in a large general population in the United States. Sci. Rep..

[14] De Meyer T., Rietzschel E.R., De Buyzere M.L., Langlois M.R., De Bacquer D., Segers P., Van Damme P., De Backer G.G., Van Oostveldt P., Van Criekinge W. (2009). Asklepios Study Investigators. Systemic telomere length and preclinical atherosclerosis: The Asklepios Study. Eur. Heart J..

[15] Rietzschel E.R., Bekaert S., De Meyer T. (2016). Telomeres and Atherosclerosis: The Attrition of an Attractive Hypothesis. J. Am. Coll. Cardiol..

[16] Bhattacharyya J., Mihara K., Bhattacharjee D., Mukherjee M. (2017). Telomere length as a potential biomarker of coronary artery disease. Indian J. Med. Res..

[17] Daniali L., Benetos A., Susser E., Kark J.D., Labat C., Kimura M., Desai K., Granick M., Aviv A. (2013). Telomeres shorten at equivalent rates in somatic tissues of adults. Nat. Commun..

[18] O’Callaghan N., Fenech M. (2011). A quantitative PCR method for measuring absolute telomere length. Biol. Proc. Online.

[19] Pańczyszyn A., Boniewska-Bernacka E., Głąb G. (2020). Telomere length in leukocytes and cervical smears of women with high-risk human papillomavirus (HR HPV) infection. Taiwan J. Obstet. Gynecol..

[20] Cawthon R. (2009). Telomere measurement by the novel monochrome multiplex quantative PCR method. Nucleic Acids Res..

[21] Olinic D.M., Spinu M., Olinic M., Homorodean C., Tataru D.A., Liew A., Schernthaner G.H., Stanek A., Fowkes G., Catalano M. (2018). Epidemiology of peripheral artery disease in Europe: VAS Educational Paper. Int. Angiol..

[22] Gallino A., Aboyans V., Diehm C., Cosentino F., Stricker H., Falk E., Schouten O., Lekakis J., Amann-Vesti B., Siclari F. (2014). Non-coronary atherosclerosis. Eur. Heart J..

[23] Anderson R., Lagnado A., Maggiorani D., Walaszczyk A., Dookun E., Chapman J., Birch J., Salmonowicz H., Ogrodnik M., Jurk D. (2019). Length-independent telomere damage drives post-mitotic cardiomyocyte senescence. EMBO J..

[24] Haycock P.C., Heydon E.E., Kaptoge S., Butterworth A.S., Thompson A., Willeit P. (2014). Leucocyte telomere length and risk of cardiovascular disease: Systematic review and meta-analysis. BMJ.

[25] Demanelis K., Jasmine F., Chen L.S., Chernoff M., Tong L., Delgado D., Zhang C., Shinkle J., Sabarinathan M., Lin H. (2020). Determinants of telomere length across human tissues. Science.

[26] Nzietchueng R., Elfarra M., Nloga J., Labat C., Carteaux J.P., Maureira P., Lacolley P., Villemot J.P., Benetos A. (2011). Telomere length in vascular tissues from patients with atherosclerotic disease. J. Nutr. Health Aging.

[27] Gutmajster E., Witecka J., Wyskida M., Koscinska-Marczewska J., Szwed M., Owczarz M., Mossakowska M., Milewicz A., Puzianowska-Kuznicka M., Zejda J. (2013). Telomere length in elderly Caucasians weakly correlates with blood cell counts. Sci. World J..

[28] Compté N., Bailly B., De Breucker S., Goriely S., Pepersack T. (2015). Study of the association of total and differential white blood cell counts with geriatric conditions, cardio-vascular diseases, seric IL-6 levels and telomere length. Exp. Gerontol..

[29] Neuner B., Lenfers A., Kelsch R., Jäger K., Brüggmann N., van der Harst P., Walter M. (2015). Telomere Length Is Not Related to Established Cardiovascular Risk Factors but Does Correlate with Red and White Blood Cell Counts in a German Blood Donor Population. PLoS ONE.

[30] Adams C.D., Boutwell B.B. (2020). A Mendelian randomization study of telomere length and blood-cell traits. Sci. Rep..

[31] Mollica L., Fleury I., Belisle C., Provost S., Roy D.C., Busque L. (2009). No association between telomere length and blood cell counts in elderly individuals. J. Gerontol. A Biol. Sci. Med. Sci..

[32] Den Elzen W.J.P., Martin-Ruiz C., von Zglinicki T., Westendorp R.G.J., Kirkwood T.B., Gussekloo J. (2011). Telomere length and anaemia in old age: Results from the Newcastle 85-plus Study and the Leiden 85-plus Study. Age Ageing.

[33] Martin-Ruiz C.M., Gussekloo J., van Heemst D., von Zglinicki T., Westendorp R.G. (2005). Telomere length in white blood cells is not associated with morbidity or mortality in the oldest old: A population-based study. Aging Cell.

[34] Mazidi M., Penson P., Banach M. (2017). Association between telomere length and complete blood count in US adults. Arch. Med. Sci..

[35] Chen W., Gardner J.P., Kimura M., Brimacombe M., Cao X., Srinivasan S.R., Berenson G.S., Aviv A. (2009). Leukocyte telomere length is associated with HDL cholesterol levels: The Bogalusa heart study. Atherosclerosis.

[36] Chen Y.F., Zhou K.W., Yang G.Z., Chen C. (2019). Association between lipoproteins and telomere length in US adults: Data from the NHANES 1999-2002. Lipids Health Dis..

[37] Rehkopf D.H., Needham B.L., Lin J., Blackburn E.H., Zota A.R., Wojcicki J.M., Epel E.S. (2016). Leukocyte Telomere Length in Relation to 17 Biomarkers of Cardiovascular Disease Risk: A Cross-Sectional Study of US Adults. PLoS Med..

[38] Hajishengallis G., Li X., Chavakis T. (2021). Immunometabolic control of hematopoiesis. Mol. Aspects Med..

[39] Baragetti A., Bonacina F., Catapano A., Danilo A., Norata G. (2021). Effect of Lipids and Lipoproteins on Hematopoietic Cell Metabolism and Commitment in Atherosclerosis. Immunometabolism.

[40] Boccardi V., Barbieri M., Rizzo M.R., Marfella R., Esposito A., Marano L., Paolisso G. (2013). A new pleiotropic effect of statins in elderly: Modulation of telomerase activity. FASEB J..

[41] Boccardi V., Paolisso G. (2014). The association between statins and telomere shortening. Clin. Lipidol..

[42] Olivieri F., Mazzanti I., Abbatecola A.M., Recchioni R., Marcheselli F., Procopio A.D., Antonicelli R. (2012). Telomere/Telomerase system: A new target of statins pleiotropic effect?. Curr. Vasc. Pharmacol..

